# Supplementary material for: Relationship between Training Factors and Injuries in Stand-Up Paddleboarding Athletes
Source: Int J Environ Res Public Health. 2021 Jan 20;18(3):880. doi: 10.3390/ijerph18030880 (PMC7908629; doi:10.3390/ijerph18030880)
Supplement: Supplementary file 1 [file ijerph-18-00880-s001.pdf]

Cuestionario SUP

Este cuestionario se ha realizado con el objetivo de conocer y poder profundizar en la tipología de las lesiones de los paddlelistas y las variables más influyentes en las mismas.

El tratamiento, la comunicación y la cesión de los datos de carácter personal de todos los sujetos participantes se ajustará a lo dispuesto en la Ley Orgánica 3/2018, de 5 de diciembre, de Protección de Datos Personales y garantía de los derechos digitales. De acuerdo a lo que establece la legislación mencionada, usted puede ejercer los derechos de oposición y cancelación de datos, para lo cual deberá dirigirse a las directoras del estudio. Las investigadoras del proyecto podrán tener acceso a los datos del voluntario. Los datos personales y la información obtenida de este estudio, con garantía de privacidad para su identidad, se conocerá sólo por las investigadoras del proyecto. Con la aceptación de este consentimiento usted autoriza la recogida, almacenamiento y análisis de sus datos solicitados, desvinculados de la identidad por un sistema de codificación doble reversible. Aquellas personas que continúen el proceso mostrarán su aceptación a las características de la investigación y asegurará su comprensión.
*Obligatorio

1. 0.- He leído y acepto las condiciones *

Marca solo un óvalo.

- Sí
- No

2. 1.- País de residencia *

____

3. 2.- Fecha de nacimiento *

____

Ejemplo: 7 de enero del 2019

4. 3.- Genero *

Marca solo un óvalo.

- Mujer
- Hombre
- Otro

5. 4.- Peso en Kgr *

6. 5.- Estatura en cm *

7. 6.- ¿Cuántos años llevas practicando SUP? *

Marca solo un óvalo.

- 1-3 años
- 3-7 años
- 8-11 años
- Más de 11 años

8. 7.- ¿Cuántos años llevas federado? *

9. 8.- ¿Compites en SUP? *

Marca solo un óvalo.

- Si
- No (sigue hasta la pregunta 11)

10. 9.1.- Si has respondido que sí: ¿En qué modalidades de SUP compites?

Selecciona todos los que correspondan.

- [ ] SUP Surf
- [ ] SUP Race
- [ ] SUP Técnica
- [ ] Ninguna

11. 9.1.1.- Si has elegido SUP Race; ¿compites en algún circuito?

Marca solo un óvalo.

- Si
- No (Ir a la pregunta 10)

12. 9.1.2.- Si has contestado que sí: ¿En cuál?

____

13. 9.1.3.- ¿En que distancia?

Marca solo un óvalo.

- Élite
- Amateur
- Otros
- Otro: _______________________________

14. 9.1.3.1. Si has escogido otro indica cual

____

15. 10.-¿Cuántas competiciones realizas al año?

Marca solo un óvalo.

- No compito
- 1-5
- 6-10
- 11-15
- 16-20
- Más de 20

16. 10.1.- ¿Cuántas de estas competiciones son internacionales?

____

17. 11.- ¿Cuál es tu lado dominante de remada? *

Marca solo un óvalo.

- Derecha
- Izquierda
- Indiferente

18. 12.- ¿Cuántos días a la semana entrenas habitualmente? *

Marca solo un óvalo.

1	2	3	4	5	6	7

19. 13.- ¿Cuántas sesiones de entrenamiento realizas habitualmente al día?

Marca solo un óvalo.

1	2	3

20. 14.- ¿Cuántas horas de media entrenas en cada sesión de entrenamiento? *

Marca solo un óvalo.

- 1h
- 1,5h
- 2h
- 2,5h
- 3h
- 3,5h
- 4h
- 4,5h

21. 15.- ¿Cuál es el volumen (tiempo) de las sesiones que más tiempo hayas entrenado? *

Marca solo un óvalo.

- 1h
- 2h
- 3h
- 4h
- 5h
- 6h
- Más de 6h

22. 16.- ¿ Complementas tu práctica de SUP con algún otro deporte? *

Marca solo un óvalo.

- Si
- No

23. 16.1.- Si la respuesta a la pregunta anterior ha sido que sí: ¿ con cuál?

____

24. 16.2.- ¿Cuántas veces a la semana?

Marca solo un óvalo.

1	2	3	4	5	6	7
◯	◯	◯	◯	◯	◯	◯

25. 17.- ¿Complementas tus sesiones de SUP con entrenamiento de fuerza en el gimnasio? *

Marca solo un óvalo.

- Si
- No (Ir a la pregunta 18)

26. 17.1.- Si la respuesta a la pregunta anterior ha sido que sí: ¿Cuántas veces a la semana?

Marca solo un óvalo.

1	2	3	4	5	6	7
⬭	⬭	⬭	⬭	⬭	⬭	⬭

27. 17.2.- ¿Cuántos meses al año?

Marca solo un óvalo.

- ⬭ 1
- ⬭ 2
- ⬭ 3
- ⬭ 4
- ⬭ 5
- ⬭ 6
- ⬭ 7
- ⬭ 8
- ⬭ 9
- ⬭ 10
- ⬭ 11
- ⬭ 12

28. 18.- ¿Complementas tus sesiones de SUP con trabajo de core? *

Marca solo un óvalo.

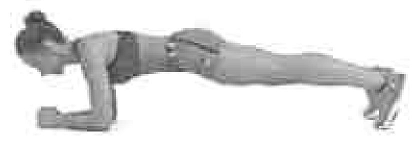

⬭ SI ⬭ No

29. 18.1.- Si la respuesta a la pregunta anterior ha sido que sí: ¿Cuántas sesiones a la semana?

Marca solo un óvalo.

1	2	3	4	5	6	7	8	9	10
⬭	⬭	⬭	⬭	⬭	⬭	⬭	⬭	⬭	⬭

30. 19.- ¿Complementas tus sesiones de SUP con ejercicios de estiramientos y flexibilidad? *

Marca solo un óvalo.

- Si
- No
- En ocasiones

31. 19.1.- Si has respondido de manera afirmativa a la pregunta anterior: ¿cuántas veces a la semana?

Marca solo un óvalo.

1	2	3	4	5	6	7
⬭	⬭	⬭	⬭	⬭	⬭	⬭

32. 19.2.- ¿En qué momento o momentos de la sesión realizas los estiramientos?

Selecciona todos los que correspondan.

- [ ] Antes
- [ ] Durante
- [ ] Después

33. 20.- ¿Cuidas tu alimentación?

Marca solo un óvalo.

- Si
- No
- En ocasiones

34. 21.- Dispones de: *

Selecciona todos los que correspondan.

- [ ] Fisioterapeuta
- [ ] Preparador físico
- [ ] Médico deportivo
- [ ] Nutricionista
- [ ] Ninguno (me entreno yo mismo)
- [ ] Otros

35. 21.1.- Si has escogido otros indica cual

____

36. 22.- ¿Cuántos remos tienes? *

Marca solo un óvalo.

- ( ) 1
- ( ) 2
- ( ) 3
- ( ) 4
- ( ) 5
- ( ) Más de 5

37. 23.- ¿Qué anchura tiene la pala que más usas?

____

38. 24.- ¿ Qué longitud tiene el remo que más usas?

____

39. 25.- ¿Cuántas tablas tienes?

Marca solo un óvalo.

- 1
- 2
- 3
- 4
- 5
- Más de 5

40. 26.- ¿ Qué longitud tiene la tabla que más usas?

____

41. 27.- ¿ Qué anchura tiene la tabla que más usas?

____

45. 4 Items

De 0- Nunca a 4- Muy a menudo

42. 45.1. En el último mes... [¿Con qué frecuencia te has sentido incapaz de controlar las cosas impor-tantes en tu vida?] *

Marca solo un óvalo.

- 0- nunca
- 1- casi nunca
- 2- de vez en cuando
- 3- a menudo
- 4- muy a menudo

43. 45.2. En el último mes... [¿Con qué frecuencia ha estado seguro sobre tu capacidad para manejar tus problemas personales?] *

Marca solo un óvalo.

- 0- nunca
- 1- casi nunca
- 2- de vez en cuando
- 3- a menudo
- 4- muy a menudo

44. 45.3. En el último mes... [¿Con qué frecuencia has sentido que las cosas te van bien?] *

Marca solo un óvalo.

- 0- nunca
- 1- casi nunca
- 2- de vez en cuando
- 3- a menudo
- 4- muy a menudo

45. 45.4. En el último mes... [¿Con qué frecuencia has sentido que las dificultades se acumulan tan-to que no puedes superarlas?] *

Marca solo un óvalo.

- 0- nunca
- 1- casi nunca
- 2- de vez en cuando
- 3- a menudo
- 4- muy a menudo

46. Tres Items

De 0 a 10

46. 46.1. Mis pensamientos son: *

Marca solo un óvalo.

	0	1	2	3	4	5	6	7	8	9	10	
CALMADO	◯	◯	◯	◯	◯	◯	◯	◯	◯	◯	◯	PREOCUPADO

47. 46.2. Mi cuerpo se siente: *

Marca solo un óvalo.

	0	1	2	3	4	5	6	7	8	9	10	
RELAJADO	◯	◯	◯	◯	◯	◯	◯	◯	◯	◯	◯	TENSO

48. 46.3. Me siento: *

Marca solo un óvalo.

	0	1	2	3	4	5	6	7	8	9	10	
SEGURO	◯	◯	◯	◯	◯	◯	◯	◯	◯	◯	◯	ASUSTADO

Todas las preguntas tendrán una opción de contestación del 1 al 5, 1 será nunca y 5 casi siempre.

49. 28.- Soy capaz de adaptarme cuando ocurren cambios *

Marca solo un óvalo.

1	2	3	4	5
◯	◯	◯	◯	◯

50. 29.- Puedo enfrentarme a cualquier cosa *

Marca solo un óvalo.

1	2	3	4	5
◯	◯	◯	◯	◯

51. 30.- Intento ver el lado divertido de las cosas cuando me enfrento con problemas *

Marca solo un óvalo.

1	2	3	4	5
◯	◯	◯	◯	◯

52. 31.- Enfrentarme a las dificultades puede hacerme más fuerte *

Marca solo un óvalo.

1	2	3	4	5
◯	◯	◯	◯	◯

53. 32.- Tengo tendencia a recuperarme pronto tras enfermedades, heridas u otras privaciones *

Marca solo un óvalo.

1	2	3	4	5
◯	◯	◯	◯	◯

54. 33.- Creo que puedo lograr mis objetivos, incluso si hay obstáculos *

Marca solo un óvalo.

1	2	3	4	5
◯	◯	◯	◯	◯

55. 34.- Bajo presión me centro y pienso claramente *

Marca solo un óvalo.

1	2	3	4	5
◯	◯	◯	◯	◯

56. 35.- No me desanimo fácilmente con el fracaso *

Marca solo un óvalo.

	1	2	3	4	5	
	◯	◯	◯	◯	◯	

57. 36.- Creo que soy una persona fuerte cuando me enfrento a los retos y dificultades de la vida *

Marca solo un óvalo.

	1	2	3	4	5	
	◯	◯	◯	◯	◯	

58. 37.- Soy capaz de manejar sentimientos desagradables y dolorosos como tristeza, temor y enfado *

Marca solo un óvalo.

	1	2	3	4	5	
	◯	◯	◯	◯	◯	

59. 38.- ¿Has sufrido o sufriste alguna molestia o lesión practicando SUP? *

Marca solo un óvalo.

◯ Si

◯ No *Salta a la pregunta 109*

I

60. 39.1.- ¿Cuándo se produjo dicha lesión? *

Marca solo un óvalo.

- ◯ En los últimos 6 meses
- ◯ En los últimos 12 meses
- ◯ En los últimos 18 meses
- ◯ En los últimos 24 meses
- ◯ Más de 24 meses

61. 39.1.1.- Si la lesión se produjo hace más de 24 meses, indique cuando;

____

62. 39.2.- Región anatómica en la que se produjo la lesión: *

Marca solo un óvalo.

- Manos
- Muñecas
- Antebrazo
- Codo
- Brazo
- Hombro
- Clavícula
- Cuello
- Columna- Espalda Baja
- Columna- Espalda Alta
- Costillas
- Pecho
- Abdomen
- Cadera
- Pelvis
- Dedos
- Muslo
- Rodilla
- Pierna
- Tobillo
- Pie
- Uñas
- Otros
- Otro: ____________________

63. 39.3.- ¿En qué lado corporal? *

Marca solo un óvalo.

- Derecho
- Izquierdo
- Centro

64. 39.4.- ¿En qué momento se produjo? *

Marca solo un óvalo.

- Calentamiento
- Entrenamiento
- Vuelta a la calma
- Competición

65. 39.5.- ¿Porque ocurrió? *

Marca solo un óvalo.

- Impacto (producido por golpe)
- Sobreuso (relacionado con el tipo y volumen de carga de entrenamiento).

66. 39.6.- ¿Qué tipo de lesión fue? *

Marca solo un óvalo.

- Nueva lesión
- Recaída

67. 39.7.- ¿Cuál fue el diagnóstico?: *

Marca solo un óvalo.

- ◯ Contusión
- ◯ Dislocación
- ◯ Esguince
- ◯ Fractura
- ◯ Herida superficial
- ◯ Irritación
- ◯ Quemadura
- ◯ Rotura muscular
- ◯ Contractura muscular
- ◯ Tendinitis
- ◯ Luxación
- ◯ Fisura
- ◯ Micro rotura muscular
- ◯ Otros
- ◯ Otro: ____________________

68. 39.8.- ¿Quién te trató la lesión? *

Selecciona todos los que correspondan.

- ☐ Médico
- ☐ Fisioterapeuta
- ☐ Entrenador
- ☐ Masajista
- ☐ Osteópata
- ☐ Otro

69. 38.8.1.- Si has escogido otro indica cual

____

70. 39.9.- ¿Cuánto tiempo estuviste sin remar por la lesión?: *

Marca solo un óvalo.

- 1-3 días
- 4-7 días
- De 1 a 2 semanas
- De 2 semanas a 1mes
- De 1 mes a 3 meses
- Más de 3 meses
- Otro: ______________________

71. 40.- ¿Has sufrido alguna otra molestia o lesión practicando SUP? *

Marca solo un óvalo.

- Si
- No *Salta a la pregunta 109*

2

72. 39.1.- ¿Cuándo se produjo dicha lesión? *

Marca solo un óvalo.

- En los últimos 6 meses
- En los últimos 12 meses
- En los últimos 18 meses
- En los últimos 24 meses
- Más de 24 meses

73. 39.1.1.- Si la lesión se produjo hace más de 24 meses, indique cuando;

____

74. 39.2.- Región anatómica en la que se produjo la lesión: *

Marca solo un óvalo.

- Manos
- Muñecas
- Antebrazo
- Codo
- Brazo
- Hombro
- Clavícula
- Cuello
- Columna- Espalda Baja
- Columna- Espalda Alta
- Costillas
- Pecho
- Abdomen
- Cadera
- Pelvis
- Dedos
- Muslo
- Rodilla
- Pierna
- Tobillo
- Pie
- Uñas
- Otros
- Otro: ____________________

75. 39.3.- ¿En qué lado corporal? *

Marca solo un óvalo.

- Derecho
- Izquierdo
- Centro

76. 39.4.- ¿En qué momento se produjo? *

Marca solo un óvalo.

- Calentamiento
- Entrenamiento
- Vuelta a la calma
- Competición

77. 39.5.- ¿Porque ocurrió? *

Marca solo un óvalo.

- Impacto (producido por golpe)
- Sobreuso (relacionado con el tipo y volumen de carga de entrenamiento).

78. 39.6.- ¿Qué tipo de lesión fue? *

Marca solo un óvalo.

- Nueva lesión
- Recaída

79. 39.7.- ¿Cuál fue el diagnóstico?: *

Marca solo un óvalo.

- Contusión
- Dislocación
- Esguince
- Fractura
- Herida superficial
- Irritación
- Quemadura
- Rotura muscular
- Contractura muscular
- Tendinitis
- Luxación
- Fisura
- Micro rotura muscular
- Otros
- Otro: __________

80. 39.8.- ¿Quién te trató la lesión? *

Selecciona todos los que correspondan.

- [ ] Médico
- [ ] Fisioterapeuta
- [ ] Entrenador
- [ ] Masajista
- [ ] Osteópata
- [ ] Otro

81. 38.8.1.- Si has escogido otro indica cual

____

82. 39.9.- ¿Cuánto tiempo estuviste sin remar por la lesión?: *

Marca solo un óvalo.

- 1-3 días
- 4-7 días
- De 1 a 2 semanas
- De 2 semanas a 1mes
- De 1 mes a 3 meses
- Más de 3 meses
- Otro: ______________________

83. 40.- ¿Has sufrido alguna otra molestia o lesión practicando SUP? *

Marca solo un óvalo.

- Si
- No *Salta a la pregunta 109*

3

84. 39.1.- ¿Cuándo se produjo dicha lesión? *

Marca solo un óvalo.

- En los últimos 6 meses
- En los últimos 12 meses
- En los últimos 18 meses
- En los últimos 24 meses
- Más de 24 meses

85. 39.1.1.- Si la lesión se produjo hace más de 24 meses, indique cuando;

____

86. 39.2.- Región anatómica en la que se produjo la lesión: *

Marca solo un óvalo.

- Manos
- Muñecas
- Antebrazo
- Codo
- Brazo
- Hombro
- Clavícula
- Cuello
- Columna- Espalda Baja
- Columna- Espalda Alta
- Costillas
- Pecho
- Abdomen
- Cadera
- Pelvis
- Dedos
- Muslo
- Rodilla
- Pierna
- Tobillo
- Pie
- Uñas
- Otros
- Otro: ________________

87. 39.3.- ¿En qué lado corporal? *

Marca solo un óvalo.

- Derecho
- Izquierdo
- Centro

88. 39.4.- ¿En qué momento se produjo? *

Marca solo un óvalo.

- Calentamiento
- Entrenamiento
- Vuelta a la calma
- Competición

89. 39.5.- ¿Porque ocurrió? *

Marca solo un óvalo.

- Impacto (producido por golpe)
- Sobreuso (relacionado con el tipo y volumen de carga de entrenamiento).

90. 39.6.- ¿Qué tipo de lesión fue? *

Marca solo un óvalo.

- Nueva lesión
- Recaída

91. 39.7.- ¿Cuál fue el diagnóstico?: *

Marca solo un óvalo.

- Contusión
- Dislocación
- Esguince
- Fractura
- Herida superficial
- Irritación
- Quemadura
- Rotura muscular
- Contractura muscular
- Tendinitis
- Luxación
- Fisura
- Micro rotura muscular
- Otros
- Otro: ____________

92. 39.8.- ¿Quién te trató la lesión? *

Selecciona todos los que correspondan.

- [ ] Médico
- [ ] Fisioterapeuta
- [ ] Entrenador
- [ ] Masajista
- [ ] Osteópata
- [ ] Otro

93. 38.8.1.- Si has escogido otro indica cual

____

94. 39.9.- ¿Cuánto tiempo estuviste sin remar por la lesión?: *

Marca solo un óvalo.

- 1-3 días
- 4-7 días
- De 1 a 2 semanas
- De 2 semanas a 1mes
- De 1 mes a 3 meses
- Más de 3 meses
- Otro: ____________________

95. 40.- ¿Has sufrido alguna otra molestia o lesión practicando SUP? *

Marca solo un óvalo.

- Si
- No *Salta a la pregunta 109*

4

96. 39.1.- ¿Cuándo se produjo dicha lesión? *

Marca solo un óvalo.

- En los últimos 6 meses
- En los últimos 12 meses
- En los últimos 18 meses
- En los últimos 24 meses
- Más de 24 meses

97. 39.1.1.- Si la lesión se produjo hace más de 24 meses, indique cuando;

____

98. 39.2.- Región anatómica en la que se produjo la lesión: *

Marca solo un óvalo.

- Manos
- Muñecas
- Antebrazo
- Codo
- Brazo
- Hombro
- Clavícula
- Cuello
- Columna- Espalda Baja
- Columna- Espalda Alta
- Costillas
- Pecho
- Abdomen
- Cadera
- Pelvis
- Dedos
- Muslo
- Rodilla
- Pierna
- Tobillo
- Pie
- Uñas
- Otros
- Otro: ____________

99. 39.3.- ¿En qué lado corporal? *

Marca solo un óvalo.

- Derecho
- Izquierdo
- Centro

100. 39.4.- ¿En qué momento se produjo? *

Marca solo un óvalo.

- Calentamiento
- Entrenamiento
- Vuelta a la calma
- Competición

101. 39.5.- ¿Porque ocurrió? *

Marca solo un óvalo.

- Impacto (producido por golpe)
- Sobreuso (relacionado con el tipo y volumen de carga de entrenamiento).

102. 39.6.- ¿Qué tipo de lesión fue? *

Marca solo un óvalo.

- Nueva lesión
- Recaída

103. 39.7.- ¿Cuál fue el diagnóstico?: *

Marca solo un óvalo.

- Contusión
- Dislocación
- Esguince
- Fractura
- Herida superficial
- Irritación
- Quemadura
- Rotura muscular
- Contractura muscular
- Tendinitis
- Luxación
- Fisura
- Micro rotura muscular
- Otros
- Otro: ____________________

104. 39.8.- ¿Quién te trató la lesión? *

Selecciona todos los que correspondan.

- [ ] Médico
- [ ] Fisioterapeuta
- [ ] Entrenador
- [ ] Masajista
- [ ] Osteópata
- [ ] Otro

105. 39.8.1.- Si has escogido otro indica cual

____

106. 39.9.- ¿Cuánto tiempo estuviste sin remar por la lesión?: *

Marca solo un óvalo.

- 1-3 días
- 4-7 días
- De 1 a 2 semanas
- De 2 semanas a 1mes
- De 1 mes a 3 meses
- Más de 3 meses
- Otro: ____________

107. 40.- ¿Has sufrido alguna otra molestia o lesión practicando SUP? *

Marca solo un óvalo.

- Si
- No

108. 40.1. Si has respondido indica cual

____

109. 41.- ¿Sufres algún dolor crónico o molestias relacionadas con el deporte del SUP? *

Marca solo un óvalo.

- Si
- No (Ir a la pregunta 42)

110. 41.1. Si has respondido si, indique donde

____

111. 41.2.- ¿Si has respondido sí, cuando se produce el dolor?

Marca solo un óvalo.

- Mientras practicas SUP
- Cuando no estás practicando SUP
- En las dos situaciones anteriores

112. 41.3.- ¿A qué crees que se debe?

Marca solo un óvalo.

- Mala preparación
- Es una vieja lesión
- Cargas de entrenamiento (demasiado entrenamiento)
- Otros motivos
- Otro: ____________________

113. 41.3.1. Si has escogido otros motivos indica cuales

Marca solo un óvalo.

- Opción 1

114. 42.- ¿Hace siempre calentamiento en tus sesiones de entrenamiento?

Marca solo un óvalo.

- Si
- No
- A veces

115. 43.- ¿Haces siempre vuelta a la calma en tus sesiones de entrenamiento?

Marca solo un óvalo.

- Si
- No
- A veces

116. 44.- ¿Realizas algún protocolo de prevención de lesiones? *

Marca solo un óvalo.

- Si
- No

117. 44.1.- Si has respondido si, indica cuantos días a la semana

Marca solo un óvalo.

- 1
- 2
- 3
- 4
- 5
- 6
- 7

Muchas gracias por su participación y aportación a este estudio.

118. Si estás interesado en recibir los resultados de esta investigación especifica tu CORREO ELECTRÓNICOa continuación

____

119. MUCHAS GRACIAS POR TU TIEMPO. Si tienes algún comentario o aclaración que desees realizar sobre el cuestionario utilice el siguiente espacio (con cualquier consulta no dude en contactarnos: arkaitz.castaneda@deusto.es)

____

Este contenido no ha sido creado ni aprobado por Google.

Google Formularios

SUP Survey

The aim of this survey is to analyse and get to know the injury typology of SUP paddlers and the variables more connected to them.

The treatment, communication and transfer of personal data of all participating subjects shall conform to the provisions of Organic Law 3/2018 of 5 December on Personal Data Protection and digital rights guarantee. In accordance with the aforementioned legislation, you may exercise your rights of disapproval and termination of data, for which you should contact the directors of the study. The investigators of the project will have access to the volunteers´ data. The personal data and the information obtained from this study, with guarantee of identity privacy, will be known only by the investigators of the project. By accepting this consent, you authorize the collection, storage and analysis of your requested data, disconnected from the identity by a double-reversible codification system. Those who continue the process will show their acceptance of the characteristics of the research and ensure their understanding.
*Obligatorio

1. 0- I have read and I accept the terms and conditions *

   Marca solo un óvalo.

   ◯ Yes
   ◯ No

2. 1.- Country of residence *

   ____

3. 2.- Date of birth *

   ____
   Ejemplo: 7 de enero del 2019

4. 3.- Gender *

   Marca solo un óvalo.

   ◯ Female
   ◯ Male
   ◯ Other

5. 4.- Weight in Kg *

____

6. 5.- Height in cm *

____

7. 6.- ¿How long have you practiced SUP? *

Marca solo un óvalo.

- ( ) 1-3 years
- ( ) 3-7 years
- ( ) 8-11 years
- ( ) More than 11 years

8. 7.- How long have you been federated? *

____

9. 8.- Do you compete in SUP? *

Marca solo un óvalo.

- ( ) Yes
- ( ) No (Go to question 11)

10. 9.1.- If your Answer was yes: In which type of SUP do you take part?

Selecciona todos los que correspondan.

- [ ] SUP Surf
- [ ] SUP Race
- [ ] SUP Technique
- [ ] None of them

11. 9.1.1.- If you chose SUP Race; Do you compete in any tour?

Marca solo un óvalo.

- ( ) Yes
- ( ) No (Go to question 10)

12. 9.1.2.- If your answer was yes, in which tour?

____

13. 9.1.3.- In which distance?

Marca solo un óvalo.

- ( ) Elite
- ( ) Amateur
- ( ) Other
- ( ) Otro: ____________________

14. 9.1.3.1. If you picked other specify which

____

15. 10.- In how many competitions do you take part during a year?

Marca solo un óvalo.

- ( ) No compito
- ( ) 1-5
- ( ) 6-10
- ( ) 11-15
- ( ) 16-20
- ( ) Más de 20

16. 10.1.- How many of them are international?

____

17. 11.- Which is your dominant paddling side? *

Marca solo un óvalo.

- Right
- Left
- Indifferent

18. 12.- How many days do you train per week? *

Marca solo un óvalo.

1	2	3	4	5	6	7

19. 13.- How many sessions do you do each day?

Marca solo un óvalo.

1	2	3

20. 14.- What is the average time that you spend during each session? *

Marca solo un óvalo.

- 1h
- 1,5h
- 2h
- 2,5h
- 3h
- 3,5h
- 4h
- 4,5h

21. 15.- What is the longest session that you have done practising SUP? *

Marca solo un óvalo.

- 1h
- 2h
- 3h
- 4h
- 5h
- 6h
- Más de 6h

22. 16.- Do you practise another sport at the same time? *

Marca solo un óvalo.

- Yes
- No

23. 16.1.- If your answer was yes, with which sport?

____

24. 16.2.- ¿How many seasons per week?

Marca solo un óvalo.

1	2	3	4	5	6	7
◯	◯	◯	◯	◯	◯	◯

25. 17.- Do you combine your SUP sessions with strength training? *

Marca solo un óvalo.

- Yes
- No (Go to question 18)

26. 17.1.- If your answer was yes, in how many days per week?

Marca solo un óvalo.

1	2	3	4	5	6	7
◯	◯	◯	◯	◯	◯	◯

27. 17.2.- How many months during the year?

Marca solo un óvalo.

- ◯ 1
- ◯ 2
- ◯ 3
- ◯ 4
- ◯ 5
- ◯ 6
- ◯ 7
- ◯ 8
- ◯ 9
- ◯ 10
- ◯ 11
- ◯ 12

28. 18.- Do you combine your SUP sessions with "core" training? *

Marca solo un óvalo.

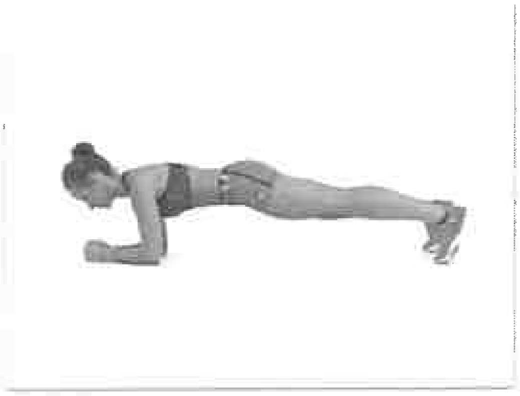

◯ Yes ◯ No

29. 18.1.- If your answer was yes, in how many sessions per week?

Marca solo un óvalo.

1	2	3	4	5	6	7	8	9	10
◯	◯	◯	◯	◯	◯	◯	◯	◯	◯

30. 19.- Do you combine your SUP sessions with stretching and flexibility exercises? *

Marca solo un óvalo.

- ◯ Yes
- ◯ No
- ◯ Sometimes

31. 19.1.- If your answer was yes, how many days per week?

Marca solo un óvalo.

1	2	3	4	5	6	7
◯	◯	◯	◯	◯	◯	◯

32. 19.2.- In which part of the session do you do stretching exercises?

Selecciona todos los que correspondan.

- ☐ Before
- ☐ During
- ☐ After

33. 20.- Do you take care of your diet?

Marca solo un óvalo.

- ◯ Yes
- ◯ No
- ◯ Sometimes

34. 21.- Have you got any of the following? *

Selecciona todos los que correspondan.

- [ ] Physiotherapist
- [ ] Physical trainer
- [ ] Sport doctor
- [ ] Nutricionis
- [ ] None of them (I train by myself)
- [ ] Other

35. 21.1.- If you chose other, specify which:

____

36. 22.- How many oars have you got? *

Marca solo un óvalo.

- ( ) 1
- ( ) 2
- ( ) 3
- ( ) 4
- ( ) 5
- ( ) Más de 5

37. 23.- ¿Which width is the blade or paddle blade that you use more often?

____

38. 24.- What is the length of the oar that you use more often?

____

39. 25.- How many boards do you have?

Marca solo un óvalo.

- ( ) 1
- ( ) 2
- ( ) 3
- ( ) 4
- ( ) 5
- ( ) Más de 5

40. 26.- What is the length of the board that you use more often?

____

41. 27.- Which width is the board that you use more often?

____

45. 4 items

From 0- never- to 4- Most often

42. 45.1. During the last month... [How often have you felt unable to control important thing of your life?] *

Marca solo un óvalo.

- ( ) 0- Never
- ( ) 1- Almost never
- ( ) 2- Occasionally
- ( ) 3- Frequently
- ( ) 4- Most often

43. 45.2. During the last month... [How often have you been certain about your ability to manage your problems?] *

Marca solo un óvalo.

- 0- Never
- 1- Almost never
- 2- Occasionally
- 3- Frequently
- 4- Most often

44. 45.3. During the last month... [How often have you felt that things were going well for you?] *

Marca solo un óvalo.

- 0- Never
- 1- Almost never
- 2- Occasionally
- 3- Frequently
- 4- Most often

45. 45.4. During the last month... [How often have you felt that difficulties were accumulated so much that you could not overcome them?] *

Marca solo un óvalo.

- 0- Never
- 1- Almost never
- 2- Occasionally
- 3- Frequently
- 4- Most often

46. 3 Items
From 0 to 10

46. 46.1. My thoughts are: *

Marca solo un óvalo.

	0	1	2	3	4	5	6	7	8	9	10	
CALM	⬭	⬭	⬭	⬭	⬭	⬭	⬭	⬭	⬭	⬭	⬭	WORRIES

47. 46.2. My body feels: *

Marca solo un óvalo.

	0	1	2	3	4	5	6	7	8	9	10	
RELAXED	⬭	⬭	⬭	⬭	⬭	⬭	⬭	⬭	⬭	⬭	⬭	TENSE

48. 46.3. I am feeling: *

Marca solo un óvalo.

	0	1	2	3	4	5	6	7	8	9	10	
CONFIDENT	⬭	⬭	⬭	⬭	⬭	⬭	⬭	⬭	⬭	⬭	⬭	SCARED

49. 28.- I am able to adapt myself when changes happen *

Marca solo un óvalo.

	1	2	3	4	5	
Never	⬭	⬭	⬭	⬭	⬭	Almost always

50. 29.- I can deal with any situation *

Marca solo un óvalo.

	1	2	3	4	5	
Never	⬭	⬭	⬭	⬭	⬭	Almost always

51. 30.- I try to see the funny side when I face problems *

Marca solo un óvalo.

	1	2	3	4	5	
Never	◯	◯	◯	◯	◯	Almost always

52. 31.- Facing difficulties can make me stronger *

Marca solo un óvalo.

	1	2	3	4	5	
Never	◯	◯	◯	◯	◯	Almost always

53. 32.- I tend to recover quickly after illness, wounds or other adversities *

Marca solo un óvalo.

	1	2	3	4	5	
Never	◯	◯	◯	◯	◯	Almost always

54. 33.- I think that I can achieve my goals even if there are obstacles *

Marca solo un óvalo.

	1	2	3	4	5	
Never	◯	◯	◯	◯	◯	Almost always

55. 34.- Under pressure I focus and I think clearly *

Marca solo un óvalo.

	1	2	3	4	5	
Never	◯	◯	◯	◯	◯	Almost always

56. 35.- I don't get discourage easily by failure *

Marca solo un óvalo.

	1	2	3	4	5	
Never	◯	◯	◯	◯	◯	Almost always

57. 36.- I consider myself strong when it comes to facing challenges and difficulties of life *

Marca solo un óvalo.

	1	2	3	4	5	
Never	◯	◯	◯	◯	◯	Almost always

58. 37.- I am able to manage painful unconfortable emotions like sadness, fear and annoyance *

Marca solo un óvalo.

	1	2	3	4	5	
Never	◯	◯	◯	◯	◯	Almost always

59. 38.- Have you ever suffered an injury while practicing SUP? *

Marca solo un óvalo.

◯ Yes

◯ No *Salta a la pregunta 109*

I

60. 39.1.- When did this injury happen? *

Marca solo un óvalo.

- ◯ In the last 6 months
- ◯ In the last 12 months
- ◯ In the last 18 months
- ◯ In the last 24 months
- ◯ More than 24 months ago

61. 39.1.1.- If the injury happened more than 24 months ago, specify when

____

62. 39.2.- Anatomical region where you suffered the injury: *

Marca solo un óvalo.

- Hands
- Wrists
- Forearm
- Elbow
- Arm
- Shoulder
- Collar bone (Clavicle)
- Neck
- Column- Lower back
- Column- Upper back
- Ribs
- Chest
- Abdomen
- Hip
- Pelvis
- Fingers or toes
- Thigh
- Knee
- Leg
- Ankle
- Foot
- Nails
- Other

63. 39.3.- In which side of your body? *

Marca solo un óvalo.

- Right
- Left
- Center (middle)

64. 39.4.- When did it happen? *

Marca solo un óvalo.

- Warm up
- Training session
- Cool down
- Competition

65. 39.5.- Why did it happen? *

Marca solo un óvalo.

- Impact (due to a bump)
- Overuse (Connected to the type and load of the work out)

66. 39.6.- Which type of injury was it? *

Marca solo un óvalo.

- New injury
- Relapse

67. 39.7.- Which was the diagnosis? *

Marca solo un óvalo.

- Contusion or bruise
- Dislocation
- Sprain
- Fracture
- Superficial wound
- Irritation
- Burn
- Torn muscle
- Muscle cramp
- Tendinitis
- Dislocation
- Fracture
- Small muscle tear
- Other

68. 39.8.- Who treated your injury? *

Selecciona todos los que correspondan.

- [ ] Doctor
- [ ] Physiotherapist
- [ ] Coach
- [ ] Massage therapist
- [ ] Osteopath
- [ ] Other

69. 38.8.1.- If you chose other, specify which

70. 39.9.- How long were you without rowing due to the injury? *

Marca solo un óvalo.

- 1-3 days
- 4-7 days
- From 1- to 2 weeks
- From 2 weeks to 1 month
- From 1 month to 3 months
- More than 3 months

71. 40.- Have you suffered another injury while practicing SUP? *

Marca solo un óvalo.

- Yes
- No *Salta a la pregunta 109*

2

72. 39.1.- When did this injury happen? *

Marca solo un óvalo.

- In the las 6 months
- In the last 12 months
- In the last 18months
- In the last 24 months
- More than 24 months ago

73. 39.1.1.- If the injury happened more than 24 months ago, specify when

74. 39.2.- Anatomical region where you suffered the injury: *

Marca solo un óvalo.

- Hands
- Wrists
- Forearm
- Elbow
- Arm
- Shoulder
- Collar bone (Clavicle)
- Neck
- Column- Lower back
- Column- Upper back
- Ribs
- Chest
- Abdomen
- Hip
- Pelvis
- Fingers or toes
- Thigh
- Knee
- Leg
- Ankle
- Foot
- Nails
- Other

75. 39.3.- In which side of your body? *

Marca solo un óvalo.

- Right
- Left
- Centre (middle)

76. 39.4.- When did it happen? *

Marca solo un óvalo.

- Warm up
- Trainning sessión
- Cool down
- Competition

77. 39.5.- Why did it happen? *

Marca solo un óvalo.

- Impact (due to a bump)
- Overuse (Connected to the type and load of the work out)

78. 39.6.- Which type of injury was it? *

Marca solo un óvalo.

- New Injury
- Relapse

79. 39.7.- Which was the diagnosis? *

Marca solo un óvalo.

- Contusion or bruise
- Dislocation
- Sprain
- Fracture
- Superficial wound
- Irritation
- Burn
- Torn muscle
- Muscle cramp
- Tendinitis
- Dislocation
- Fracture
- Small muscle tear
- Other

80. 39.8.- Who treated your injury? *

Selecciona todos los que correspondan.

- [ ] Doctor
- [ ] Physiotherapist
- [ ] Coach
- [ ] Massage therapist
- [ ] Osteopath
- [ ] Other

81. 38.8.1.- If you chose other, specify which

____

82. 39.9.- How long were you without rowing due to the injury? *

Marca solo un óvalo.

- 1-3 days
- 4-7 days
- From 1- to 2 weeks
- From 2 weeks to 1 month
- From 1 month to 3 months
- More than 3 months

83. 40.- Have you suffered another injury while practicing SUP? *

Marca solo un óvalo.

- Yes
- No *Salta a la pregunta 109*

3

84. 39.1.- When did this injury happen? *

Marca solo un óvalo.

- In the las 6 months
- In the last 12 months
- In the last 18months
- In the last 24 months
- More than 24 months ago

85. 39.1.1.- If the injury happened more than 24 months ago, specify when

____

86. 39.2.- Anatomical region where you suffered the injury: *

Marca solo un óvalo.

- Hands
- Wrists
- Forearm
- Elbow
- Arm
- Shoulder
- Collar bone (Clavicle)
- Neck
- Column- Lower back
- Column- Upper back
- Ribs
- Chest
- Abdomen
- Hip
- Pelvis
- Fingers or toes
- Thigh
- Knee
- Leg
- Ankle
- Foot
- Nails
- Other

87. 39.3.- In which side of your body? *

Marca solo un óvalo.

- Right
- Left
- Centre (middle)

88. 39.4.- ¿En qué momento se produjo? *

Marca solo un óvalo.

- Warm up
- Training session
- Cool down
- Competition

89. 39.5.- Why did it happen? *

Marca solo un óvalo.

- Impact (due to a bump)
- Overuse (Connected to the type and load of the work out)

90. 39.6.- Which type of injury was it? *

Marca solo un óvalo.

- New Injury
- Relapse

91. 39.7.- Which was the diagnosis? *

Marca solo un óvalo.

- Contusion or bruise
- Dislocation
- Sprain
- Fracture
- Superficial wound
- Irritation
- Burn
- Torn muscle
- Muscle cramp
- Tendinitis
- Dislocation
- Fracture
- Small muscle tear
- Other

92. 39.8.-Who treated your injury? *

Selecciona todos los que correspondan.

- [ ] Doctor
- [ ] Physiotherapist
- [ ] Coach
- [ ] Massage therapist
- [ ] Osteopath
- [ ] Other

93. 38.8.1.- If you chose other, specify which

94. 39.9.- How long were you without rowing due to the injury? *

Marca solo un óvalo.

- ( ) 1-3 days
- ( ) 4-7 days
- ( ) From 1- to 2 weeks
- ( ) From 2 weeks to 1 month
- ( ) From 1 month to 3 months
- ( ) More than 3 months

95. 40.- Have you suffered another injury while practicing SUP? *

Marca solo un óvalo.

- ( ) Yes
- ( ) No *Salta a la pregunta 109*

4

96. 39.1.- When did this injury happen? *

Marca solo un óvalo.

- ( ) In the las 6 months
- ( ) In the last 12 months
- ( ) In the last 18months
- ( ) In the last 24 months
- ( ) More than 24 months ago

97. 39.1.1.- If the injury happened more than 24 months ago, specify when

____

98. 39.2.- Anatomical region where you suffered the injury: *

Marca solo un óvalo.

- Hands
- Wrists
- Forearm
- Elbow
- Arm
- Shoulder
- Collar bone (Clavicle)
- Neck
- Column- Lower back
- Column- Upper back
- Ribs
- Chest
- Abdomen
- Hip
- Pelvis
- Fingers or toes
- Thigh
- Knee
- Leg
- Ankle
- Foot
- Nails
- Other

99. 39.3.- In which side of your body? *

Marca solo un óvalo.

- Right
- Left
- Centre (middle)

100. 39.4.- When did it happen? *

Marca solo un óvalo.

- Warm up
- Training session
- Cool down
- Competition

101. 39.5.- Why did it happen? *

Marca solo un óvalo.

- Impact (due to a bump)
- Overuse (Connected to the type and load of the work out)

102. 39.6.- Which type of injury was it? *

Marca solo un óvalo.

- New Injury
- Relapse

103. 39.7.- Which was the diagnosis? *

Marca solo un óvalo.

- Contusion or bruise
- Dislocation
- Sprain
- Fracture
- Superficial wound
- Irritation
- Burn
- Torn muscle
- Muscle cramp
- Tendinitis
- Dislocation
- Fracture
- Small muscle tear
- Other

104. 39.8.- Who treated your injury? *

Selecciona todos los que correspondan.

- [ ] Doctor
- [ ] Physiotherapist
- [ ] Coach
- [ ] Massage therapist
- [ ] Osteopath
- [ ] Other

105. 39.8.1.- If you chose other, specify which

____

106. 39.9.- How long were you without rowing due to the injury? *

Marca solo un óvalo.

- 1-3 days
- 4-7 days
- From 1- to 2 weeks
- From 2 weeks to 1 month
- From 1 month to 3 months
- More than 3 months

107. 40.- Have you suffered another injury while practicing SUP? *

Marca solo un óvalo.

- Yes
- No

108. 40.1. If your answer was yes, specify which

____

109. 41.- Do you suffer any chronic pain or discomfort connected to SUP sport? *

Marca solo un óvalo.

- Yes
- No (Go to question 42)

110. 41.1. If your answer was yes, specify which part of your body

____

111. 41.2.- If your answer was yes, specify when?

Marca solo un óvalo.

- While I am practicing SUP
- When I am not practicing SUP
- In the previous two situations (always)

112. 41.3.- Why do you think it happens?

Marca solo un óvalo.

- Poor preparation
- It is an old injury
- Training load
- Other reasons

113. 41.3.1. If you chose "other reasons" specify which

____

114. 42.- Do you always warm up in your training sessions?

Marca solo un óvalo.

- Yes
- No
- Sometimes

115. 43.- Do you always cool down in your training sessions?

Marca solo un óvalo.

- Yes
- No
- Sometimes

116. 44.- Do you follow any injury prevention protocol? *

Marca solo un óvalo.

- Yes
- No

117. 44.1.- If your answer was yes, specify how many days per week

Marca solo un óvalo.

- 1
- 2
- 3
- 4
- 5
- 6
- 7

Thanks a lot for your participation and support in this study

118. If you are interested in receiving the results of this research, write down your EMAIL ADDRESS in the following gap

____

119. THANK YOU VERY MUCH FOR YOUR TIME. If you need any explanation or clarification about this survey, use the following gap (with any other doubt, do not hesitate to write to: arkaitz.castaneda@deusto.es)

____

Este contenido no ha sido creado ni aprobado por Google.

Google Formularios
